# Application of Ovarian Cancer Organoids in Precision Medicine: Key Challenges and Current Opportunities

**DOI:** 10.3389/fcell.2021.701429

**Published:** 2021-08-02

**Authors:** Jiani Yang, Shan Huang, Shanshan Cheng, Yue Jin, Nan Zhang, Yu Wang

**Affiliations:** ^1^Department of Obstetrics and Gynecology, School of Medicine, Renji Hospital, Shanghai Jiao Tong University, Shanghai, China; ^2^Shanghai Key Laboratory of Gynecologic Oncology, Shanghai, China

**Keywords:** organoid, ovarian cancer, preclinical tumor models, precision medicine, three-dimensional platform

## Abstract

Ovarian cancer (OC) is the leading cause of death among gynecologic malignances. Over the past decades, human-derived models have advanced from monolayer cell cultures to three-dimensional (3D) organoids that could faithfully recapitulate biological characteristics and tumor heterogeneity of primary tissues. As a complement of previous studies based on cell lines or xenografts, organoids provide a 3D platform for mutation–carcinogenesis modeling, high-throughput drug screening, genetic engineering, and biobanking, which might fulfill the gap between basic research and clinical practice. Stepwise, cutting-edge bioengineering techniques of organoid-on-a-chip and 3D bioprinting might converge current challenges and contribute to personalized therapy. We comprehensively reviewed the advantages, challenges, and translational potential of OC organoids. Undeniably, organoids represent an excellent near-physiological platform for OC, paving the way for precision medicine implementation. Future efforts will doubtlessly bring this innovative technique from bench to bedside.

## Introduction

Ovarian cancer (OC) is one of the most lethal gynecologic malignant tumors, with 13,940 deaths and 21,750 new cases estimated for 2020 in the United States (Siegel et al., 2020). Because of the lack of early symptoms or manifestations, over 70% of OCs were diagnosed at an advanced stage, leading to a poor 5-year survival rate under 40% (Ebell et al., 2016). Moreover, OC is a heterogeneous carcinoma with a broad spectrum of entities, among which epithelial type accounts for approximately 50–70% OC cases and can be mainly divided into type I and type II and borderline subgroups (Koshiyama et al., 2014). Type I tumors (accounting for 28% OC cases), mainly including low-grade serous (LGS), clear cell (CCC), mucinous (MC), and endometrioid (END) carcinomas, have various frequently mutated genes of KRAS, PTEN, BRAF, and CTNNB1 (Kopper et al., 2019). Type II tumors (accounting for 57% OC cases) include high-grade serous ovarian cancer (HGSOC), the most common type accounting for 70–80% of OC mortalities, with frequent gene mutations in TP53 (96%) and BRCA1/2 (20%) (Cancer Genome Atlas Research Network, 2011). Considering the significant heterogeneity, after conventional “one-size-fits-all” treatment of cytoreductive surgery and platinum-taxane chemotherapy, almost 80% of OC patients suffer cancer progression or recurrence, which drives attention to the personalized “one dose, one patient” therapy (Webb and Jordan, 2017). To overcome the issue, new anticancer compounds are in trial for OC. Representatively, for those OC cases deficient in the homologous recombination (HR) repair, a specific DNA repair defect often caused by inactivation of BRCA1/2, poly (ADP-ribose) polymerase (PARP) inhibitors are practical to improve prognosis, although it can still be ineffective among other OC patients without HR deficiency (Lord and Ashworth, 2017). Moreover, up until now, many therapeutic agents working in cancer models finally failed in clinical settings, owing to intolerant effects (DiMasi et al., 2013). For instance, multitargeted antiangiogenic agents (such as Pazopanib) could have toxicities including hypertension, liver toxicity, neutropenia, diarrhea, fatigue, and thrombocytopenia, which might limit their application in OC (du Bois et al., 2014). In this regard, developing promising preclinical models is of great urgency.

Throughout the last decade, organoids spring up as an independent complement of conventional preclinical tumor models of two-dimensional (2D) cell lines or patient-derived xenografts (PDXs). Organoids are 3D constructs that initiate from organ-restricted adult stem cells (ASCs), pluripotent embryonic stem cells (ESCs), or synthetic induced pluripotent stem cell (iPSC) counterparts of ESCs, and then be embedded in a 3D matrix with a cocktail of growth and signaling factors in order to grow with high efficiencies into an organotypic structure (Clevers, 2016). Especially, tumor-derived organoids are one type cultured from resected tumor biopsy tissues, from which tumor cells can be dissociated through mechanical and enzymatic digestion for organoid generation (Drost and Clevers, 2018). However, the efficiency of iPSC-based organoid development might depend on specific oncogenic mutations and cancer type, which could result in selection for tumor subclones and loss of genetic heterogeneity (Papapetrou, 2016). We focus on OC organoids directly derived from patients, which appears more practical than to involve an intermediate iPSC step. OC organoids can closely recapitulate primary tissue in terms of structures and functions, as promising mini-replicas of cancer tissues (Maru et al., 2019a). Moreover, OC organoids can maintain a genomic landscape and tumor heterogeneity of original cells to a large extent, thus serving as an amazing *in vitro* tool to discover the underlying mechanism of cancer processes in preclinical research (Lohmussaar et al., 2020a). Despite these strengths, OC organoids also have several limitations. OC organoids still have hurdles to duplicate the internal tumor microenvironment (TME) without real-time spatiotemporal control over biochemical and biophysical factors. Moreover, considering that the procedure of organoid development and components of culture mediums vary a lot among different researchers (Table 1), detailed experiment assays for OC organoids should be standardized by experts.

**TABLE 1:** A brief overview of experimental conditions and key findings of studies on OC organoid cultures (Hill et al., 2018; Kopper et al., 2019; Maru et al., 2019b; Hoffmann et al., 2020; Maenhoudt et al., 2020; Nanki et al., 2020).

Features	Hill et al., 2018	Kopper et al., 2019	Hoffmann et al., 2020	Maru et al., 2019b	Maenhoudt et al., 2020	Nanki et al., 2020
ECM	Matrigel	BME	Matrigel	Matrigel	Matrigel	Matrigel
Culturing medium	Advanced DMEM/F12	Advanced DMEM/F12	Advanced DMEM/F12	Advanced DMEM/F12	Advanced DMEM/F12	Advanced DMEM/F12
**Growth factors and other components of the medium**						
HEPES	1:100	10 mM	10 mM	1:100	1:100	2 mM
Penicillin–Streptomycin	1x	1x	1x	1x	1x	200 U/ml
Primocin	(−)	0.20%	(−)	(−)	(−)	(−)
GlutaMAX	1x	1x	1x	1x	1x	1x
B27	1x	1x	1x	(-)	1x	1x
N2	(−)	(−)	1x	(−)	1x	(−)
Nicotinamide	10 mM	10 mM	1 mM	(−)	5 mM	(−)
EGF	50 ng/ml	5 μg/ml	10 ng/ml	50 ng/ml	50 ng/ml	(−)
FGF2	10 ng/ml	(−)	(−)	(−)	(−)	50 ng/ml
FGF10	10 ng/ml	10 ng/ml	(−)	(−)	(−)	(−)
HGF	(−)	(−)	(−)	(-)	10 ng/ml	(−)
A83-01	500 nM	0.5 μM	(−)	(−)	0.25 μM	500 nM
N-acetylcysteine	1.25 mM	1.25 mM	(−)	(−)	1.25 mM	1 Mm
Noggin	100 ng/ml	1%	(−)	100 ng/ml	100 ng/ml	100 ng/ml
WNT3A	(−)	20%	(−)	(−)	(−)	20%
R-spondin1	100 ng/ml	10%	(−)	250 ng/ml	50 ng/ml	1 μg/ml
p38 inhibitor	10 μM	(−)	0.5 μM	(−)	1 μM	(−)
17-β Estradiol	(−)	100 μM	(−)	(−)	10 nM	(−)
Hydrocortisone	250 mg/ml	500 ng/ml	(−)	(−)	(−)	(−)
Y-27632	10 mM	5 μM	9 μM	10 μM	10 μM	10 μM
IGF1	(−)	(−)	(−)	(−)	20 ng/ml	100 ng/ml
BMP2	(−)	(−)	10 ng/ml	(−)	(−)	(−)
Heregulinβ-1	(−)	25 μg/ml	(−)	(−)	(−)	(−)
Leu15-Gastrin I	(−)	(−)	(−)	(−)	(−)	10 nM
NRG1	(−)	(−)	(−)	(−)	50 ng/ml	(−)
Organoid formation efficiency	80–90% (solid tumor), 100% (ascitic/pleural fluid)	65%	∼30% efficiency	83%	56%	80% (28/35)
Onset of organoids formation	7–10 days	1–2 weeks	NA	NA	2–4 weeks	1–3 weeks
Expansion	6–30 passages	15–30 passages	>1 year	>2 passages	NA	NA
Purpose	Predict DNA repair inhibitor response in OC organoids	Develop OC organoids to capture intra- and interpatient heterogeneity	Develop OC organoids in low-Wnt environment for long-term growth	Modified Matrigel bilayer organoid culture (MBOC) as an effective preclinical model	OC organoids as powerful tools for drug screening; identify NRG1 as a key factor of OC organoids	OC organoids capture genomic profiles; applied for drug sensitivity and resistance testing

*BME, basement membrane extract; DMEM/F12, Dulbecco’s modified Eagle medium/nutrient mixture F12; HEPES, 4-(2-hydroxyethyl)-1-piperazine-ethane-sulfonic acid; FGF2, fibroblast growth factor-2; FGF10, fibroblast growth factor-10; BMP2, bone morphogenetic protein 2.*

In this review, we aimed to give a general overview of the critical challenges and opportunities of this revolutionary patient-derived model for OC research in a push toward personalized medicine. We highlighted the great potential of patient-derived OC organoids in mainly four vital biomedical research directions: tumor modeling, drug screening, genetic engineering, and biobanking. Considering the technique challenges faced by this innovative technique, we also indicated the recent efforts exerted by scientists to promote the clinical implementation of OC organoids in the realm of bioengineering, through the advanced methods of organoid-on-a-chip and 3D bioprinting.

## Current Human-Derived Models in OC

**FIGURE 1:**
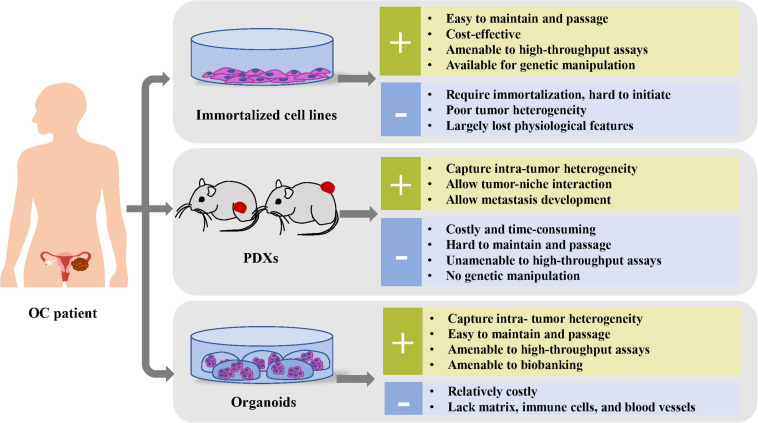
Comparison of the advantages and disadvantages of preclinical cancer models, including immortalized cell lines, PDXs, and organoids.

Currently, human-derived preclinical models for OC mainly consist of immortalized cell lines, PDXs, and organoids, among which, cell lines are most widely used. To date, the organoids approach, a robust 3D *in vitro* system that faithfully replicates primary tumor, has drawn great attention among oncologists, with its potential to rectify most shortcomings of other preclinical models (Figure 1).

### Conventional 2D Cell Lines

As a traditional preclinical model, monolayer cell cultures derived from patients could offer a promising *in vitro* platform for cell biology research, genomic manipulation, and high-throughput screenings. Moreover, cell lines are easy to maintain and passage at a relatively low cost (Drost and Clevers, 2018). However, the success rate to establish new cell lines from patient-derived tissues is limited and unpredictable (Verschraegen et al., 2003). Moreover, these cancer cell lines largely failed to model the TME, because of their lack of interactions between tumor cells and immune cells, stromal cells, and extracellular matrix (ECM) (McMillin et al., 2013). Additionally, these 2D cell line cultures consist of a mostly homogeneous tumor cell population because of long-term clonal selection (Domcke et al., 2013).

Ovaries are composed of various cell types, including granulosa and theca cells, which have diverse functions of reciprocal cross-talk and cell–cell interactions (Song et al., 2019). Therefore, isolating and culturing the cell population in a conventional monolayer dish might distort findings recapitulating physiology *in vivo*. A recent analysis of OC cultures indicated that conventional OC cell lines of SKOV-3 and A2780, derived from HGSOC, lack several main hallmarks of this subtype, including extensive genomic instability and TP53 mutations, which could limit the validation and clinical relevance of numerous previous studies (Domcke et al., 2013; Papp et al., 2018). Thus, conventional monolayer cell cultures might so far fail in precise tumor modeling of OC.

### PDX Models

The PDX model, an immune-deficient mice approach transplanted by tissue fragments of fresh human tumor, shows the tremendous clinical application in the aspect of pharmacodynamics and pharmacokinetics evaluation within a relatively *in vivo* environment comprising both stroma and host cells (including fibroblasts, immune cells, endothelial cells, and blood cells) (Depreeuw et al., 2015). Promising PDX models have already been developed for major OC subtypes, among which HGSOC has the highest success rate (Liu et al., 2017). Bankert et al. (2011) reported that PDXs could reliably recapitulate tumor progression, ascite formation, and metastasis in the OC development process. Based on their ability to maintain heterogeneity of original tumors, PDXs were used to exploit for the response toward platinum-based chemotherapy (Ricci et al., 2014) and targeted therapies, including HER2-targeted antibodies for HER2-positive PDXs (Harris et al., 2019) and PARP inhibitors for BRCA-deficient xenografts (George et al., 2017).

Although PDXs show remarkable preservation for histological characteristics and molecular heterogeneity of original tumor in previous OC research (Aparicio et al., 2015), the genomic stability over multiple generations has also been recently questioned by a study that reported accumulated genetic changes of copy number alterations over passages (Ben-David et al., 2017). Additionally, because of the slow speed of tumor growth, low engraftment rates, and high costs, the PDX platform application is limited for high-throughput screening and large-scale clinical applications (Lohmussaar et al., 2020a). Moreover, PDXs based on immunocompromised mice are limited in experiments of immunomodulatory compounds (Chen and Mellman, 2017). Alternatively, the humanized mice, generated through transplantation of human immune cells or hematopoietic stem cells into immune-deficient mice, could reconstitute the human immunologic environment, although with challenging technique concerns (Buque and Galluzzi, 2018; Li et al., 2020).

### Organoid Cultures

#### General Overview for Organoids

**FIGURE 2:**
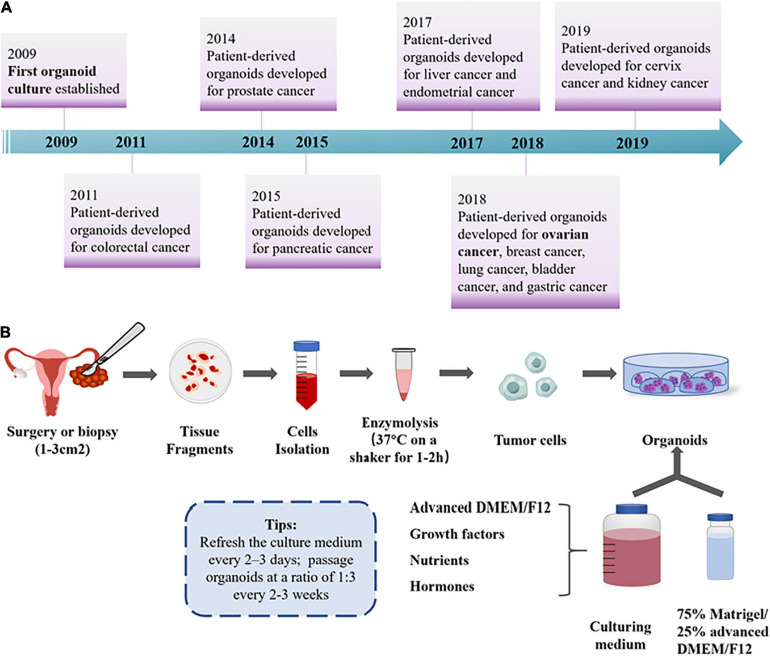
General overview for OC organoids. **(A)** Timeline for patient-derived organoid development until now. **(B)** The workflow of OC organoid culture development. Formation of organoids was initiated by mechanical and enzymatic digestion of primary tumor tissue, followed by seeding cells into the fundamental ECM and culturing medium with various growth factors for long-term maintenance (Hill et al., 2018; Kopper et al., 2019).

Organoids are 3D cultures derived from primary tissues, ASCs, or direct-differentiated pluripotent stem cells. Sato et al. (2009) claimed that LGR5^+^ -marked murine intestinal stem cells could proliferate infinitely through the organoid, a mini-organ *in vitro* composed of epithelial cells. Right now, organoid models have been gradually established for various tumor types, including lung (Kim et al., 2019), prostate (Karthaus et al., 2014), breast (Harper et al., 2016), liver (Nuciforo et al., 2018), gastric (Wang et al., 2014), colorectal (Matano et al., 2015), pancreatic (Huang et al., 2015), kidney (Grassi et al., 2019), bladder (Mullenders et al., 2019), endometrium (Boretto et al., 2019), ovarian (Hill et al., 2018; Kopper et al., 2019), and cervix (Maru et al., 2019a) cancer in various culture conditions (Figure 2A). The tumor organoid approach is initiated by mechanical and enzymatic digestion of primary tumor tissue, followed by embedding cells into a specific matrix (such as Matrigel) and culturing medium, supplemented with a cocktail of growth factors and hormones for long-term maintenance (Hill et al., 2018; Figure 2B). During the past decade, 3D organoid models that aim to mimic numerous vital features from the primary organ *in vitro* have drawn attention among oncologists. Based on the accuracy to recapitulate histological features, genomic characteristics, and intra- and interpatient heterogeneity of original tumor tissues, OC organoids have been applied with great clinical potential (Maenhoudt et al., 2020), in contrast with previous cancer models of cell lines or PDXs (Figure 1).

#### Ovarian Organoids Replicate Primary Tissue Characteristics

OC organoids aim to recapitulate the morphological heterogeneity of primary tumor tissue, including histological and grade variations (Kopper et al., 2019). In previous studies, scientists claimed that organoids generated from OC tissues, ascitic, and pleural fluids of metastatic OC held promise to maintain the features even after several generations (Maenhoudt et al., 2020). Condello et al. (2015) demonstrated that SKOV3 cells formed a glandular configuration, IGROV1 formed a branching one, whereas A2780 cells display dense multicellular spheres in related organoids. As for patient-derived organoids of various histological OC subtypes, borderline OC organoids have a cystic phenotype; HGSOC has either a cystic or a dense structure, which varies in cellular cohesiveness and circularity with great heterogeneity; whereas all others form dense formations with multiple lumens (Kopper et al., 2019). Meanwhile, Maru et al. (2019b) also reported faithful duplication of histological features and tumor heterogeneity within ovarian organoids derived from various subtypes, including HGSOC, MC, and END OCs. Preservation of tissue-specific molecular and proliferation (Ki67) markers has been noted, besides histological concordance (including breach of the basement membrane, pleiomorphic nuclei, and disorganized epithelium) of OC organoids with primary tissue (Turco et al., 2017).

Moreover, malignant organoids also aim to maintain genetic heterogeneity and the mutational fingerprint of primary tumor tissue. Recent single-cell DNA sequencing analysis based on 56 organoid lines developed from 32 OC cases demonstrated that OC organoids reliably preserve genomic mutations identified in BRAF, KRAS, PTEN, CDKN2A/B, TP53, and RB1, even after prolonged passaging (Kopper et al., 2019). Notably, researchers indicated that the long-term time frame required for the generation of organoid lines might lead to *in vitro* clonal selection (Drost et al., 2016). To mitigate the concerns, the team of Hill developed short-term HGSOC organoids, which were grew within 7–10 days of plating and performed experiments within one to two passages. Through whole-exome sequencing (WES) analysis, they demonstrated that the short-term organoids could retain copy number alterations, including single nucleotide variation, copy number alterations, and structural variation of parent tumors (Hill et al., 2018).

#### Microenvironment of Organoid Cultures

The TME includes tumor-secreted ECMs and tumor supporting cells, which form complex interactions with cancer cells, influencing the efficacy of cancer cell-targeting therapies. Compared with traditional cultures for monolayer cell lines, organoid cultures have additional components of ECM substitutes and specific culturing media to preserve constant stem cells and support long-term passages in various malignancies, which could partly mimic the TME components. The ECM represents a non-cellular organic component, which could provide biochemical signals essential for cell survival and biostructural scaffolding for vessels, epithelium, and stroma (Walker et al., 2018). Consistently, Nallanthighal et al. (2019) demonstrated that ECM could serve as a niche for cancer stem cells while regulating epithelial–mesenchymal transition (EMT), self-renewing properties, and tumor progression. Currently, lots of ECM biomaterials have already been used in organoid cultures, including mouse-derived substitutes (Matrigel), synthetic polymers [polyethylene glycol (PEG) or polyglutamic acid (PGA)], and natural biopolymers (hyaluronic acid or collagen) (Semertzidou et al., 2020). However, Matrigel, a solubilized basement membrane preparation rich in ECM proteins (mainly includes laminin, collagen IV, heparan sulfate proteoglycans, and entactin/nidogen), which is extracted from Englebreth–Holm–Swarm (EHS) mouse sarcoma, was commonly used to generate OC organoids with great potentials to increase cell stemness (Chang et al., 2020). Different TMEs are associated with various ECM compositions, with an impact on ovarian carcinogenesis through cell–matrix interaction, a vital element that should be taken into consideration for designing organoid cultures (Semertzidou et al., 2020). As for OC, Cho et al. (2015) demonstrated that the ECM dysregulation (including collagen fibril thickening with perpendicular orientation, hyaluronan accumulation, ECM protein cross-linking, loss of decorin, and upregulation of fibronectin, tenascin-X, and tenascin-C) is attributed to tumor stiffness, which leads to local invasion, distant migration, and tumor progression. Moreover, Sittadjody et al. (2017) used alginate hydrogel as an anchor matrix to design multilayered microcapsules and successfully recapitulate native cell–cell interactions between ovarian granulosa and theca cells in this specific ECM. Furthermore, by culturing cells in various concentrations of collagen hydrogels (ranging from 1 to 7% weight/volume), they proved that cell survival, oocyte maturation, follicle development, and sex hormone production were associated with modifications in collagen hydrogel density and elasticity, indicating the significance of ECM properties on function and phenotype maintenance of 3D culture for OC (Joo et al., 2016).

Beyond ECM, tumor cells are exposed to biochemical signals, including growth factors, nutrients, hormones, and antibodies, transduced through arterial vasculature supplying them. In order to achieve long-term prosperity, researchers try to maintain organoids in various specific mediums, which could replicate *in vivo* signals to address nutritional demands (Table 1). Since previous research suggested signal pathways including Wnt, Notch, and BMP as indispensable regulators for intracellular processes, their components are included in media for long-term cell prosperity of OC organoids (Kopper et al., 2019). Controversially, another research team demonstrated that the Wnt pathway’s inhibition could promote the growth of organoids and the expression of CD133, a stem cell marker expressed on the surface OC cells. Interestingly, they also indicated that the generation of HGSOC organoids depended on active BMP signaling, while healthy fallopian tube organoids required BMP suppression by Noggin (Hoffmann et al., 2020). Moreover, the addition of neuregulin-1 (NRG1) into the medium could promote a significant increase in proliferating (Ki67^+^) cells as well as organoid formation efficiency and passage ability (Maenhoudt et al., 2020). Besides, other growth factors and signaling molecules, including epidermal growth factor (EGF), insulin-like growth factor 1 (IGF1) and hepatocyte growth factor (HGF), nicotinamide, transforming growth factor-beta (TGF-β) inhibitor, NRG1, ROCK inhibitor, and p38 mitogen-activated protein kinase inhibitor (p38i, SB203580) were also added into the culture medium to increase OC organoid proliferation and thrive, while the Rho kinase inhibitor, Y-27632, is vital for organoid initiation (Hoffmann et al., 2020).

Nevertheless, besides matrix and growth factors, cancer cells in organoids are also exposed to vital mechanophysical aspects, including spatiotemporal oxygen gradients (Al-Ani et al., 2018), pH maintenance, and mechanotransduction forces (Michl et al., 2019), which should also be under consideration in OC organoid development. For instance, a recent research indicated that the differential concentrations and gradients of oxygen could influence gene expression of cancer cells by ECM organization (Wulftange et al., 2019). Accordingly, scientists exerted to realize dynamic monitor of biochemical factors (especially oxygen gradients) in organoids, through developing techniques including electron parametric resonance oximetry, microfluidic cell models, and dispersible oxygen biosensors (Lesher-Perez et al., 2017; Drost and Clevers, 2018). Moreover, considering that most intracellular biological processes are sensitive to acidity–alkalinity, researchers also tried to monitor and control dynamic pH value in organoid cultures, via appropriate buffering regime (including physiological CO_2_/HCO_3_- and non-volatile buffers), in order to realize long-term maintenance (Michl et al., 2019). As for local tissue mechanics, Mok et al. (2020) developed thermoresponsive material microgels to optically measure elasticity in live multicellular spheroids, with high-resolution sensors to map the spatial distribution of rigidity inside the 3D constructs.

## Clinical Application of Organoids in OC

**FIGURE 3:**
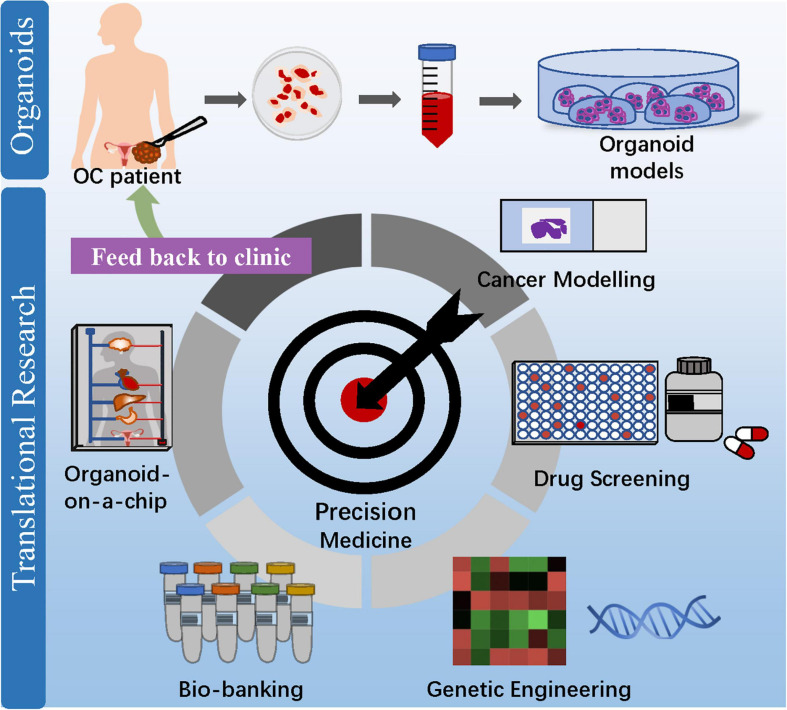
Clinical application of OC organoids from bench to bedside. Patient-derived organoids can be used for cancer modeling, drug screening, biobanking, and organoid-on-a-chip, which aim at maximizing drug efficacy and enabling precision medicine to benefit OC patients.

From bench to bedside, patient-derived OC organoids can be used for cancer modeling, drug screening, biobanking, and organoid-on-a-chip (Figure 3). Undeniably, OC organoids are expected to stand their ground to enable personalized therapy and maximize treatment efficacy, paving the way for precision medicine application.

### Modeling Carcinogenesis and Progression

Cancer is a gradual accumulation of mutations of tumor-driving genes, making it vital to get insight into mutational processes active during tissue homeostasis and tumorigenesis (Stratton et al., 2009). Recently, advanced techniques to generate healthy organoids with genetic stability over long periods open up new horizons to discover the underlying mechanism of carcinogenesis (Behjati et al., 2014). Through genetic engineering techniques, models generated by the viral introduction of cDNA or shRNA into healthy organoids can be subjected to in-depth investigation for carcinogenesis (Huch et al., 2015). Previous studies claimed that multistep processes of cancer initiation could be formed as subcutaneous tumors in nude mice via lentiviral gene transduction into murine organoids, similar to earlier *in vivo* studies of the intestine (Onuma et al., 2013), hepatobiliary (Ochiai et al., 2019), and lung cancers (Sato et al., 2017). However, as for OC, the mutation landscape is highly diverse, which raises the urgency for powerful tools to study the mutational accumulation process of various OC subtypes.

To date, several studies tried to explore the mutational processes active in carcinogenesis and progression, based on OC organoids. A research team developed a stable shRNA knockdown model of PTEN, p53, and retinoblastoma protein (RB) in fallopian tube organoids derived from patients, under a low-Wnt environment. They demonstrated that the triple-knockdown would result in the procarcinogenic phenotype of increased DNA double-strand breaks, phosphorylated Akt/cyclin E1 upregulation, and nuclear atypia, although insufficient for complete malignant transformation, with the elements of differentiation potential to ciliated phenotype and apicobasal polarity. Stepwise, researchers also conducted subcutaneous and orthotopic transplantation of OC organoids into mice and developed invasive tumors retaining both histological traits and biomarkers of original OC tissue, so as to verify the conclusions *in vivo* (Hoffmann et al., 2020). Moreover, Kopper and colleagues electroporated patient-derived organoids with plasmids targeting both TP53 and RB1 genes. They indicatedthat stable clones with the double-knockout could result in morphological OC progression (including increased cell shedding into the organoid lumen and transition from cystic to dense cell configurations), meanwhile the RNA-seq clustering analysis pertinent to corresponding mutations (Kopper et al., 2019). Another recent research highlighted the importance of the NRG1/ERBB pathway in OC progression by testing multiple culture medium components and identifying NRG1 as a critical factor in maximizing OC organoid development and growth (Maenhoudt et al., 2020). These studies highlighted the great potential of organoids to investigate the role of specific genetic mutations both in OC initiation and progression.

Recently, scientists have tried to combine the cutting-edge technique of organoids with CRISPR-Cas9, an accurate gene-editing method, to investigate the roles of specific germline mutations in malignances, although with limited researches in the realm of OC (Yu et al., 2019). Researchers developed a radiation modifier screen using the CRISPR interference (CRISPRi) technique for gene knockdown and then modeled invasive growth of glioma through human brain organoids composed of mature neural cell types, concluding that antisense oligonucleotides targeting lncGRS-1 could sensitize tumor cells to radiation therapy and decrease tumor growth (Liu et al., 2020). As reported, a study used CRISPR-Cas9 genome-edited hPSC as the source for organoids and then revealed the role of ferroptosis in DGUOK mutant mtDNA depletion syndrome (Guo et al., 2021). Although not the case for OC modeling, these studies provided hints to direct hPSC differentiation through CRISPR-Cas9 for OC researches. Lohmussaar et al. (2020b) conducted CRISPR-Cas9 gene editing to introduce Pten, Nf1, Brca1, and Trp53, the mutations commonly found in HGSOC, into murine ovarian organoids, which successfully generate tumors resembling human tumors with similar molecular signatures. They also demonstrated that epithelia could give rise to ovarian tumors with malignant pathology, supporting the dual-origin hypothesis of HGSOC (Lohmussaar et al., 2020b). Similarly, through engineering mouse fallopian tube epithelial organoids with both CRISPR-Cas9 mutagenesis and lentiviral gene transduction, Zhang et al. (2021) successfully generated HGSOC organoids exhibiting mutational combinations found in patients, including HR-deficient (Trp53^–/–^, Brca1^–/–^, and MycOE), HR-proficient (Trp53^–/–^, Ccne1OE, Akt2OE, and KrasOE), and unclassified (Trp53^–/–^, Pten^–/–^, and Nf1^–/–^) mutations, with differences in *in vitro* properties (differentiation, proliferation, and “secretome”), copy number aberrations, and tumorigenicity. Considering that those studies were based on murine ovarian organoids, future researches are still needed to validate the results in patient-derived OC organoids through the CRISPR-Cas9 technique.

### Drug Response and Screening

Drug screening on conventional cell lines provided rough insights into gene–drug associations with a relatively low reflection of original tumor tissues, which might contribute to a high failure rate of clinical trials for newly discovered drugs (Kamb, 2005). Alternatively, patient-derived tumor organoids have superior application for drug testing and great potential for high-throughput screening of anticancer drugs, with better morphological and genetic recapitulation from primary tumors (Sachs et al., 2018). So far, drug screens on organoids have yielded promising results for OC, stratifying individuals into specific treatment regimens. Considering that a majority of OC patients suffered chemotherapy or radiotherapy resistance with no or minimal therapeutic benefit, which might lead to a poor prognosis (Webb and Jordan, 2017), the effort to develop a reliable model predicting patient responsiveness to treatment and making a precise clinical decision is of great urgency to improve survival (Semertzidou et al., 2020).

Owing to the inherent molecular heterogeneity for OC, various drug responses of organoids have been reported with limited overlap. To explore the potential of patient-derived organoids for evaluating drug responses in preclinical settings, a recent study tested the effects of various standard chemotherapies used in the clinic (carboplatin, paclitaxel, gemcitabine, and doxorubicin) and revealed distinct drug sensitivities on different established OC organoid lines. Especially, researchers proved that nutlin-3, a recently explored therapeutic agent targeted for TP53 wild-type OC (Zanjirband et al., 2017), had different efficiency according to patient’s TP53 status, indicating that organoids derived from a TP53 wild-type tumor were more sensitive to nutlin-3 (Maenhoudt et al., 2020). Another research team cultured patient-derived organoids to assess gene–drug associations and revealed that organoids had great potential for drug-screening assays, concluding that the combination of paclitaxel and carboplatin failed to refine efficacy among most OC organoids, compared with carboplatin monotherapy (Jabs et al., 2017). Besides platinum/taxane drugs that are commonly used in OC treatment protocols, Kopper et al. (2019) also included drugs targeting the PI3K/AKT/mTOR pathway (pictilisib, alpelisib, AZD8055, and MK2206), PARP (niraparib), gemcitabine, and the tyrosine kinase Wee1 (adavosertib). They demonstrated differential drug responses of individual organoid lines by drug screening assays. For instance, organoids shown to be sensitive to PARP inhibitors were more likely to be HR deficient, which resembles the findings in clinical trials (Kopper et al., 2019). Stepwise, a current study, based on 36 whole-genome-characterized organoids derived from 23 OC patients with known clinical histories, evaluated the capability of organoids to display both inter- and intrapatient heterogeneity of clinical drug response and functional consequences toward chemotherapy/targeted drugs. The results indicated that drug screening of OC organoids could identify high responsiveness to at least one drug for 88% of patients, providing insights into drug response for individuals, as a complement to genetic testing (de Witte et al., 2020). Especially, OC organoids can also be xenografted orthotopically or subcutaneously into immunodeficient mice and recapitulate drug sensitivity of the original tumor with a success rate of 25–100%, enabling *in vivo* drug sensitivity assays to further improve clinical decision making (Phan et al., 2019).

Of note, immunotherapy, as a novel component of precision treatment, aims to employ patients’ own immune system to kill cancer cells (Kenny et al., 2007). To date, breakouts in immune checkpoint therapy (including CTLA-4 inhibitors and PD-1/PD-L1) highlight the application of reliable preclinical models to evaluate immune responses (Darvin et al., 2018). A previous study demonstrated that organoids have intrinsic limitations with the absence of stroma, immune cells, and blood vessels, steering a trend toward the coculture systems incorporating additional microbial and cellular elements (Ohlund et al., 2017). Recently, several studies demonstrated the ideal potentials of the complicated organoid-based coculture system in researches focused on immunotherapy, although they have not been validated in OC organoids (Workman et al., 2017). Researchers established coculture procedures in which hemocytes are cultured together with organoids, revealing the application of testing cytotoxicity of donors’ T cells into patient-derived tumor organoids for specific immunotherapy (Nozaki et al., 2016; Stronen et al., 2016). Another study reported that intraepithelial lymphocytes (IELs) in a coculture with intestinal epithelial organoids could be maintained and expanded for 2 weeks while retaining functionality, in the presence of interleukin-2 (IL-2), IL-7, and IL-15 (Nozaki et al., 2016). Nevertheless, recently developed thymus organoids might provide a more physiological *in vitro* setting in order to maintain and expand those cancer-specific T lymphocytes with excellent efficiency in OC researches for immunotherapy drug screening (Sato and Clevers, 2015; Stronen et al., 2016).

Besides testing drug efficiency, organoids can also be generated from healthy tissue, allowing for specific drug screening for target tumor cells without harming healthy ones to reduce toxicities in patients (Aboulkheyr Es et al., 2018). The majority of antitumor drug failures in clinical trials were caused by intolerant side effects, including hepatotoxicity, nephrotoxicity, and cardiotoxicity (Ballet, 1997). Katsuda et al. (2017) developed hepatic organoids on purpose to provide a promising preclinical platform for hepatotoxicity testing. Encouragingly, the hepatic organoids could express cytochrome P450, an enzyme commonly mediating hepatotoxicity, at a nearly physiological level (Huch et al., 2015).

### Organoid Biobanks

Living biobanks are repositories for cancer organoids of diverse subtypes and grades, which can be cryopreserved and passaged with immediate accessibility, high cost-effectiveness, and proliferative capacity *in vitro*. Up to date, gynecological oncologists have exerted great efforts on OC organoid biobank generation by developing various organoid lines with high efficiency. For example, a study reported efficient derivation and long-term expansion of 56 OC organoid lines from 32 patients, which represented all main subtypes of OC, with histological and genomic features of pertinent lesions (Kopper et al., 2019). The research team of Hoffmann successfully generated 15 organoid lines from 13 to 45 patients (∼30% efficiency) under the low-Wnt conditions for long-term *in vitro* propagation. They passaged the OC organoids every 10–20 days for more than 5 months before cryopreservation and indicated that organoids could be expanded to multiwell drug screening without noticeable changes in morphology or expansion potential, even after multiple cycles of thawing and freezing. Accordingly, OC organoids are suited for biobanks to explore individual therapy resistance and drug sensitivity, moving toward more efficient (patient-tailored) OC therapy (Hoffmann et al., 2020). Moreover, Maenhoudt et al. (2020) generated 13 organoid lines out of 39 samples, with moderate derivation efficiency (36% for HGSOC patients, 44% for all OC patients), by adding the key factor of NRG1 into the culture medium to maximize OC organoid development and growth. They also further demonstrated that these organoid lines could show patient tumor-dependent morphology characteristics (including dense, cystic, and low-cohesive) and faithfully recapitulated marker expressions and mutational landscape of the parent tumor, even after being cryopreserved (Maenhoudt et al., 2020). Hill et al. (2018) developed 34 organoid lines (one to four tumor sites per subject) from 23 OC patients, with a generation rate of 80–90%. Besides cytologic and morphologic features, these organoid lines also had genomes match for corresponding tumors, analyzed through WES. Recently, a combined effort was undertaken by the US National Cancer Institute (NCI), the UK Welcome Trust Sanger Institute and the Human Cancer Models Initiative (HCMI), and Cancer Research UK to generate a global-accessible biobank of various cancer cell cultures, including OC, available for research purposes (Drost and Clevers, 2018).

## Challenges and Perspectives in OC Organoid Models

### General Overview of Key Challenges and Limitations

Despite rapid innovation in organoids, there are still several shortcomings of this innovative technique. Firstly, most organoids comprise only the epithelial layer without physiological microenvironment, including mesenchyme, nervous system, muscular layer, and immune cells (Jabs et al., 2017). Although the current organoid technique still does not allow real-time spatiotemporal control over biochemical and biophysical factors, the coculture system could partly duplicate the internal TME by incorporating additional microbial and cellular elements (Morgan et al., 2017). For instance, in a recent study of iPSC-derived tissue engineering, scientists successfully developed a functional enteric nervous system in organoid cultures, which demonstrated the potential to create more complex organoid-based coculture structures (Workman et al., 2017). Moreover, in order to faithfully mimic the complexity of human organs *in vitro*, researchers claimed that organoid culture systems should include biomaterials (such as hydrogels) to emulate biomechanical and biochemical properties in ECM. For instance, Curvello et al. (2021) investigated collagen-nanocellulose (COL-NC) hydrogels as a thermoresponsive matrix with a synergic bioactive effect to support organoid initiation and growth. Compared with the traditional matrix (Matrigel), hydrogels could provide an affordable, sustainable, and performant thermoresponsive matrix for OC organoids. Secondly, currently, the components and process of organoid protocols vary a lot among different researchers (Table 1), which might raise doubts about the consistency of results. As for OC, Hoffmann et al. (2020) developed OC organoids under the low-Wnt conditions for long-term *in vitro* propagation, while another research team reported another protocol, involving the additional component of NRG1, with beneficial effects for OC organoid formation efficiency and passage ability (Maenhoudt et al., 2020). For scalability and accuracy of conclusions based on OC organoids, future attempts should also be placed on standard experiment assays with the consensus among experts (Dutta et al., 2017). Thirdly, considering the specific culture medium, including the ECM (such as Matrigel) and supplemental factors (e.g., growth factors and hormones), organoids are more costly compared to monolayer cell cultures, which might limit their application (Nagle et al., 2018).

### Converge Challenges With Advanced Bioengineering Techniques

#### 3D Bioprinting of Organoids

The 3D bioprinting technique could be used to develop sophisticated organoid models based on sequential processes involving tissue imaging, tissue construction design, and the final step of inkjet, microextrusion, or laser bioprinting (Murphy and Atala, 2014). By delivering primary cells into macromolecules and biomaterials, scientists could develop lifelike 3D bioprinting organoids amenable to transplantation, although still with some ethical concerns (Ong et al., 2017). Laronda et al. (2017) built a functional bioprosthetic ovary into sterilized mice using 3D bioprinting of microporous hydrogel scaffolds, among which follicle-seeded scaffolds become highly vascularized, and reproductive function was preserved in the bioprosthetic ovary when implanted in surgically sterilized mice. These findings indicated that the 3D bioprinting scaffold pore architecture was a critical variable in manufactured scaffold design for functional tissue engineering of organoids, through the phenomenon that 90° scaffold organoids had open porosity that limited follicle–scaffold interaction (Laronda et al., 2017). Moreover, as for OC, another study team developed a high-throughput cell printing system upon a 3D coculture model of OC cells and normal fibroblasts micropatterned on Matrigel through the 3D bioprinting technique. Within a spatially controlled microenvironment (cell–cell distance and cell density) in a high-throughput manner, OC cells remained viable during printing and proliferated following patterning, which could realize the miniaturization of an established organoid model and provide a tool for high-throughput OC drug screening through 3D bioprinting (Xu et al., 2011).

#### Organoid-on-a-Chip

By combining organoid with organ-on-a-chip, the cutting-edge bioengineering technology marks the dawn of the organoid-on-a-chip era, developing culture models with interdisciplinary convergence in more realistic experimental conditions (Takebe et al., 2017). The organoid-on-a-chip, a term forged since 2019, is a microfabricated platform with living cells, ECM, and microstructures that mimic organs (in the aspect of the cellular population, cytoarchitecture, and function), featured by capacities of continuous flow perfusion culture and precise microenvironment control (Park et al., 2019). For instance, to develop a lung-on-a-chip with mechanically active alveolar–capillary interface, Huh et al. (2010) cultured microvascular endothelial cells and human alveolar epithelial cells in the microdevice with cyclic suction and physiological flow in side chambers to reproduce rhythmic breathing movements. Moreover, they also described how this technique could be adapted to construct other human organ chips, indicating great potentials for the application organoid-on-a-chip in OC (Huh et al., 2013).

Moreover, the human microphysiological system could be developed via the integration of multiple mini-organs into various microchambers interconnected through microfluidic channels (Park et al., 2019). Of note, by incorporating various organoids together on a chip, scientists could study cancer multiorgan metastasis and microenvironment interactions on a unique platform. Current findings support the generous application of organoid-on-a-chip as a powerful *in vitro* approach with organ–organ integration. Skardal et al. (2017) successfully developed a multiple-organoid model by combining 3D printed heart and liver organoids with micro-engineered lung tissues in a modular fashion and perfusing them with a common media in a closed loop on a chip. This micro-engineered heart–lung–liver model revealed the cardiotoxicity of a chemotherapeutic drug (bleomycin), based on the cytokine-mediated cross-talk between the heart and lung organoids on the chip. Another research team established a microfluidic system that imitates the female reproductive tract and endocrine loops among organoids for the ovary, fallopian tube, uterus, cervix, and liver sustained circulating flow (Xiao et al., 2017). Accordingly, although the organoid-on-a-chip technique has not been validated in OC researches yet, future utilization of this approach might allow direct evaluation of the underlying mechanism of multiorgan OC metastasis and related endocrine loops with great potential.

## Conclusion

Precision medicine, with genomic-based drug response prediction for individuals, could enable better decision making in OC. Over the past decades, human-derived models have advanced from traditional monolayer cultures and PDXs to the innovative organoids that better recapitulate key features and functions of original tumors. Patient-derived organoids could serve as a biobank providing constant cell material for research in the realms of cancer modeling, drug testing, and genetic engineering. Oncology research based on organoids could play a vital role in carcinogenic pathways and targeted genetic manipulation, tailoring the most appropriate therapy for individuals. Although several challenges need to be addressed, the advanced techniques of 3D bioprinting and organoid-on-a-chip might further promote the translational application of organoids. Conclusively, organoids appear as an ideal cell culture model for precision medicine in OC, with their structural and functional consistency *in vivo* settings. With the rapid pace of technological advancements, organoids hold enormous potential to accurately model human tumors, ushering in a new era of precision medicine.

## Author Contributions

JY, SH, and YW designed the study. JY and SH drafted the manuscript. SC, YJ, and YW participated in the editing and revision of the manuscript. JY drawn all the figures. All authors have reviewed and approved the final manuscript prior to submission.

## Conflict of Interest

The authors declare that the research was conducted in the absence of any commercial or financial relationships that could be construed as a potential conflict of interest.

## Publisher’s Note

All claims expressed in this article are solely those of the authors and do not necessarily represent those of their affiliated organizations, or those of the publisher, the editors and the reviewers. Any product that may be evaluated in this article, or claim that may be made by its manufacturer, is not guaranteed or endorsed by the publisher.

## Abbreviations

FGF: fibroblast growth factor
LGSOC: low-grade serous ovarian cancer
FLCs: follicle-like cells.
